# Predicting Dementia With Prefrontal Electroencephalography and Event-Related Potential

**DOI:** 10.3389/fnagi.2021.659817

**Published:** 2021-04-13

**Authors:** Dieu Ni Thi Doan, Boncho Ku, Jungmi Choi, Miae Oh, Kahye Kim, Wonseok Cha, Jaeuk U. Kim

**Affiliations:** ^1^Korea Institute of Oriental Medicine, Daejeon, South Korea; ^2^Korean Convergence Medicine, University of Science and Technology, Daejeon, South Korea; ^3^Human Anti-Aging Standards Research Institute, Uiryeong-gun, South Korea; ^4^Korea Institute for Health and Social Affairs, Sejong, South Korea

**Keywords:** dementia, Alzheimer’s disease, electroencephalography, electrophysiology, event-related potential, Mini-Mental Status Examination

## Abstract

**Objective**: To examine whether prefrontal electroencephalography (EEG) can be used for screening dementia.

**Methods**: We estimated the global cognitive decline using the results of Mini-Mental Status Examination (MMSE), measurements of brain activity from resting-state EEG, responses elicited by auditory stimulation [sensory event-related potential (ERP)], and selective attention tasks (selective-attention ERP) from 122 elderly participants (dementia, 35; control, 87). We investigated that the association between MMSE and each EEG/ERP variable by using Pearson’s correlation coefficient and performing univariate linear regression analysis. Kernel density estimation was used to examine the distribution of each EEG/ERP variable in the dementia and non-dementia groups. Both Univariate and multiple logistic regression analyses with the estimated odds ratios were conducted to assess the associations between the EEG/ERP variables and dementia prevalence. To develop the predictive models, five-fold cross-validation was applied to multiple classification algorithms.

**Results**: Most prefrontal EEG/ERP variables, previously known to be associated with cognitive decline, show correlations with the MMSE score (strongest correlation has *|r|* = 0.68). Although variables such as the frontal asymmetry of the resting-state EEG are not well correlated with the MMSE score, they indicate risk factors for dementia. The selective-attention ERP and resting-state EEG variables outperform the MMSE scores in dementia prediction (areas under the receiver operating characteristic curve of 0.891, 0.824, and 0.803, respectively). In addition, combining EEG/ERP variables and MMSE scores improves the model predictive performance, whereas adding demographic risk factors do not improve the prediction accuracy.

**Conclusion**: Prefrontal EEG markers outperform MMSE scores in predicting dementia, and additional prediction accuracy is expected when combining them with MMSE scores.

**Significance**: Prefrontal EEG is effective for screening dementia when used independently or in combination with MMSE.

## Introduction

Dementia is a clinical syndrome that comprises a group of neurodegenerative disorders related to cognitive decline that influence memory, language presentation, social abilities, and executive functions, et cetera (McKhann et al., 2011; DSM-5). With the progression of cognitive decline, dementia patients gradually experience memory deficits, communication disorders, and difficulty performing activities of daily living and eventually become fully dependent on caregivers (Chertkow et al., 2013). Alzheimer’s disease (AD) is the most common cause of dementia, representing 60%–70% of cases. Other common causes of dementia include cerebrovascular disease, Lewy Bodies disease, and frontotemporal dementia (World Health Organization, 2020).

Aging is the major risk factor for dementia, which has a prevalence of approximately 97% in population aged 65 years and above (Alzheimer’s Association Report, 2020). The increasing world population and life expectancy have led to a rapid increase in the number of dementia patients, which is estimated to reach 82 million people worldwide by 2030 (World Health Organization, 2020). A substantial burden on social care and degradation of quality of life may follow. Furthermore, the deaths attributed to AD have positioned this condition as the fifth leading cause of death globally, causing 122,019 deaths in 2018 alone (Alzheimer’s Association Report, 2020).

Although no known treatment is highly effective for any type of dementia, combined therapeutic tools which are available to mitigate the after effects of cognitive impairment, especially during the early stages of these diseases (Robinson et al., 2015; Tisher and Salardini, 2019). Moreover, the effectiveness of early therapeutic interventions can be increased to achieve disease modification when neuronal degeneration has not yet begun (Sperling et al., 2011; Tisher and Salardini, 2019). As the disease progresses, neurons accumulate abnormal proteins, such as beta-amyloid and tau proteins, and exhibit mitochondrial dysfunction and calcium homeostasis dysregulation (Niedowicz et al., 2011; Kocahan and Doan, 2017; Farooqui, 2019). In the later stages, the brain of the patient presents neuroinflammation and irreversible synaptic loss, leading to neuronal death and brain tissue damage (Niedowicz et al., 2011; Kocahan and Doan, 2017; Farooqui, 2019).

Early detection of neuronal damage in the brain that enables both timely therapeutic intervention to manage the symptoms and adequate preparation of patients and caregivers. Early prediction of dementia is possible when the underlying disease is defined with tangible biomarkers. Recently, the national institute on aging and the Alzheimer’s association proposed an AD research framework using diagnostic biomarkers that are standardized in terms of beta-amyloid deposition, pathologic tau, and neurodegeneration, representing a shift from syndrome to biological constructions (Sperling et al., 2011; Jack et al., 2018). Beta-amyloid plaques and neurofibrillary tau tangles uniquely characterize AD among various neurodegenerative disorders that may progress to dementia (McKhann et al., 2011; Jack et al., 2018). Although these biomarker profiles are stated as core neuropathologic changes for defining AD and related terms in the research framework, they remain incomplete and inadequate for clinical practice (Jack et al., 2018). Furthermore, they are clinically accessible only at advanced hospitals and are frequently costly, invasive, and time consuming. Therefore, the development of cheap, fast, and easily accessible diagnostic and screening tools is needed (Humpel, 2011; Zvěřová, 2018).

At present, the most widely used tool for screening dementia is the Mini-Mental Status Examination (MMSE), which exhibits good internal consistency and concurrent validities (Boban et al., 2012; Baek et al., 2016). MMSE has been used as a clinical index to evaluate global cognitive performance with five domains: orientation, registration, attention and calculation, memory, and language (Folstein et al., 1975). Each MMSE domain functionally reflects neural activities by specific cognitive processing mechanisms. The noninvasive methods of EEG or ERPs can electrically record these neural activities. Several studies have validated the correlation between MMSE scores and EEG/ERPs variables. For instance, the study of Garn et al. (2014) explained 36%–51% of the variances associated with quantitative EEG markers by using MMSE scores and exhibited a strong correlation between MMSE scores and event-related potential (ERP) face-name encoding task. There was a significant negative correlation between MMSE scores with the temporal theta to alpha ratio, with *r* = −0.69 in AD group (Meghdadi et al., 2021). Significant correlations of MMSE with EEG beta activity were also observed (Lees et al., 2016) along with P300 latency (Tanaka et al., 1998; Lee et al., 2013). Notably, MMSE scores were effectively correlated with prefrontal EEG slowing biomarkers, as indicated from one of our previous publications (Choi et al., 2019).

Meta-analysis showed that using the MMSE alone yielded a pooled accuracy of 85%–87% for sensitivity and 82%–90% for specificity to screen dementia (cutoff value of 24–25); after adjusting for education level, the sensitivity and specificity were 97% and 70%, respectively (Creavin et al., 2014). In another review of the conversion from mild cognitive impairment (MCI) into AD dementia, the MMSE provided 27%–89% pooled sensitivity with 32%–90% specificity (Arevalo-Rodriguez et al., 2015). Although these meta-analyses have demonstrated a moderate to high accuracy of the MMSE for screening dementia, the cross-validation approach has frequently been neglected; this has led to questions regarding the overfitting of the selected models. In the medical sciences, a cross-validation approach is being increasingly adopted to obtain an unbiased prediction accuracy with high reliability (Wong and Yeh, 2020). Even though MMSE is the most prevalent screening tool for dementia, it suffers from some limitations such as barriers due to language or educational background, the learning effect, or low sensitivity in the early stage of cognitive decline (Scazufca et al., 2009; Duff et al., 2012; Carnero-Pardo, 2014; Gross et al., 2018).

Electroencephalography (EEG) may overcome or supplement the limitations of conventional screening tools such as the MMSE for the early detection of dementia, as it is non-invasive, relatively inexpensive, and portable, while allowing repeated measurements with none or minimal learning effects (Ben-David et al., 2011). Numerous studies have demonstrated that resting-state EEG biomarkers or event-related potential (ERP) components obtained from EEG signals are reliable for distinguishing dementia from normal controls or other neurological disorders. For instance, by using quantitative EEG features with artificial neural networks, the classification of MCI from elderly normal individuals produced 95.87% sensitivity and 91.06% specificity (Rossini et al., 2008) and a classification model between AD and MCI achieved 94.10% accuracy (Buscema et al., 2007). Further, 92.2% accuracy was obtained for an Area Under the Receiver Operating Characteristic curve (AUROC) of 0.965 by using the cognitive data cluster of the Consortium to Establish a Registry for the Alzheimer’s Disease (CERAD) neuropsychological battery, MMSE, and clinical dementia rating; however, in combination with a quantitative EEG analysis of the absolute band power at rest, 95.3% accuracy was achieved with an AUROC of 0.983 when distinguishing AD patients from non-AD persons (Fonseca et al., 2011). In addition, the N200 ERP component can identify memory changes better than MMSE (Papaliagkas et al., 2008).

With the recent advances in hardware and signal processing techniques, EEG systems with fewer channels have become emerging research topics as they can improve the simplicity and convenience of data acquisition and analysis in clinical environments. For instance, single-channel EEG signals have been tested for the detection of MCI, reaching 87.9% accuracy by using a support vector machine with leave-one-out cross-validation (Khatun et al., 2018). Similarly, single-channel EEG features, such as the power spectrum or amplitude, and ERP features (e.g., latency) have been used to distinguish early AD from normal controls, while reaching 81.90% accuracy (Cho et al., 2003). More recently, Choi et al. (2019) used the prefrontal EEG signals (channels Fp1 and Fp2 in the 10–20 system) and obtained a correlation of up to 0.757 in the regression model to predict the MMSE score for older individuals.

In this study, we intended to examine whether prefrontal EEG can be used for screening dementia. First, we examined the correlations between the MMSE score and selected EEG/ERP variables. Second, we compared the distributions of selected variables between the dementia and cognitively normal persons. Third, we estimated the associations of these variables with dementia using logistic regression. Finally, we developed prediction models for dementia by combining variables from resting-state EEG, sensory event-related potential (ERP), and selective-attention ERP results. We compared the model prediction accuracies with and without the MMSE score and demographic information, and we verified the applicability of the models by performing a double cross-validation test.

## Materials and Methods

### Subjects

From September to October 2017, 155 elderly individuals from four health centers (two geriatric hospitals: sites 1 and 2; two public health centers: sites 3 and 4) were recruited for this study. The participants, aged 50 years or older, were located in Uiryeong County, Korea. This observational study was performed as part of the Brain Aging Map Project, a community welfare project conducted in Uiryeong County. Four clinical research nurses were trained to operate EEG systems and other devices and performed participation scheduling, data acquisition, and result consultations. County dwellers were recruited through phone calls, brochures, flyers, and poster advertisements.

The individuals participated voluntarily for approximately, 90 min to measure global cognitive decline [MMSE-DS, a Korean version of the MMSE (Tae et al., 2010)], geriatric depression [KGDS, a Korean version of the Geriatric Depression Scale (GDS; Kim et al., 2001; Bae and Cho, 2004)], and EEG/ERP examinations, among others. All the confirmed demented patients were clinically diagnosed in the current centers by their clinicians or by previous medical exams conducted from other hospitals. Medical records including the diagnostic details for dementia patients were not provided in this study. Despite this limitation, dementia patients were confirmed according to a standardized diagnostic guideline, according to “Clinical practice guideline for dementia by Clinical Research Center for Dementia of South Korea” (Bon et al., 2011). This so-called CREDOS CPG was established in 2011, and offers clinical standards for AD dementia and vascular dementia in South Korea; dementia is diagnosed by comprehensive assessment of dementia, which includes history-taking, neurological examinations, neuropsychological tests, physical evaluation, brain imaging, and laboratory tests. The Diagnostic and Statistical Manual of Mental Disorders IV (DSM- IV; American Psychiatric Association, 2013) was used for the dementia criteria (Bell, 1994), and the International Statistical Classification of Diseases and Related Health Problems 10th edition (ICD-10) was used to classify the disease stage (World Health Organization, 1992). All the normal individuals were recruited from public health centers with the assumption that they showed no evidence of dementia. The following individuals and subjects were excluded from the study: those who had a meal or performed intensive physical exercise within 1 h before beginning the experiments; those who had insufficient sleep (<4 h) during the previous night; those with physical abnormalities that impeded adequate EEG electrode placement; and those not apt for the study as assessed by the clinical research nurses.

**Figure 1:**
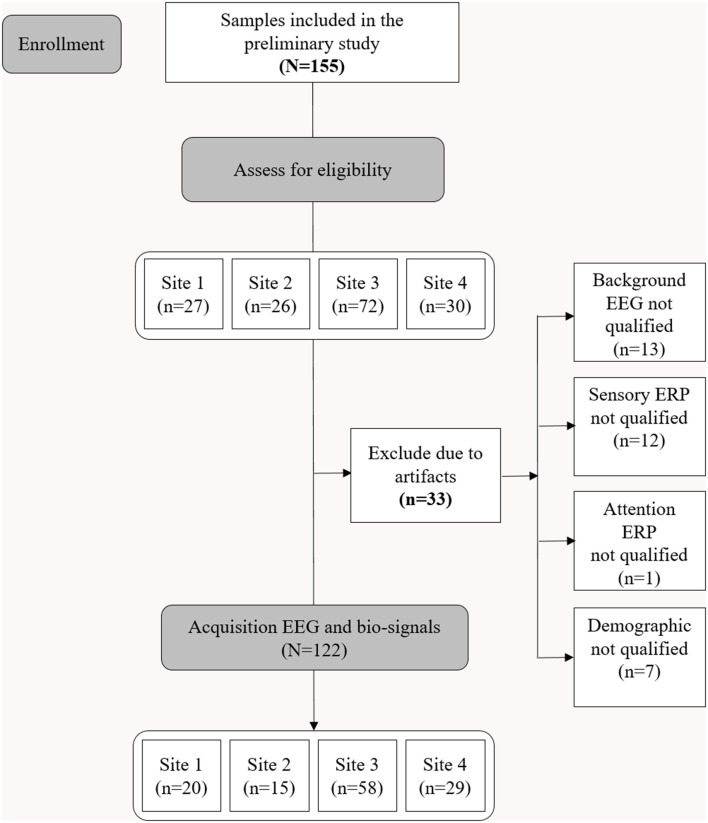
Consolidated Standards of Reporting Trials (CONSORT) diagram illustrating enrollment and exclusion criteria for this study.

Consent was obtained after providing complete descriptions about the purpose of the study to the participants or their caregivers. The study protocol was approved by the Institutional Review Board of the Korea Institute of Oriental Medicine (KIOM; approval number: I-1807/007-003). The study was performed in accordance with the Declaration of Helsinki. Figure 1 shows the consolidated standards of reporting trials (CONSORT) diagram corresponding to this study.

The demographic data, including age, sex, education level, comorbidities, and current treatments, were obtained from the participants. Subsequently, they underwent the MMSE-DS, KGDS, and EEG/ERP experiments.

### EEG/ERP Acquisition and Experiments

The brain activity was noninvasively recorded via EEG at two prefrontal monopolar scalp electrodes (channels Fp1 and Fp2) according to the International 10-20 system, with the right earlobe electrode serving as a reference. The EEG system used was the NeuroNicle FX2 (LAXTHA, Daejeon, South Korea) with band-pass filtering from 3–43 Hz and input voltages of ±393 μV (input noise below 0.6 μVrms). The signals passed through an infinite impulse response, including Butterworth highpass and lowpass filters with cutoff frequencies of 2.6 and 43 Hz, respectively. In addition, a bandstop filter was set between 55 and 65 Hz. All the EEG electrode contact impedances were maintained below 10 kΩ. The data were digitized in continuous recording mode at a 250 Hz sampling frequency and a 15-bit resolution (Choi et al., 2019). To eliminate muscle and eye movement artifacts and monitor sleepiness in the subjects, qualified operators inspected the individuals and EEG traces during the recordings. The operator guided the participants to remain comfortably seated with their eyes closed and alerted them whenever signs of behavioral or EEG drowsiness were detected. Thirty-three subjects were excluded from the study due to noise, artifacts, and incomplete demography information (Figure 1; Choi et al., 2019).

Electroencephalography (EEG) signals from the participants were acquired while they remained seated in an upright position under three sequential conditions: (1) spontaneous brain activity to establish background EEG signals in a resting state for 5 min (resting-state EEG); (2) sensory-evoked potentials (sensory ERP) for 8 min; and (3) a selective attention task to acquire the corresponding ERPs (selective-attention ERP) for 5 min. All participants were tested for auditory hearing ability before operating the experiments.

To elicit the sensory ERP, each participant was instructed to avoid motion while perceiving eight intonations from auditory stimuli at 125, 250, 500, 750, 1,500, 2,000, 3,000, and 4,000 Hz. The sequence of intonation was allocated by a pseudo-random function, in which the same intonation was not provided consecutively over the 480 stimuli presented. The pseudorandomized eight intonations function as non-repeated stimuli, which helps to avoid the sensory adaptation effect and therefore maintain the response sensitivity. Sensory adaptation leads to the attenuation of neuronal responsiveness over time after the sensory neurons are exposed to a repeated stimulation (Pérez-González and Malmierca, 2014). Another reason for selecting eight intonations, lies in the fact that hearing loss due to aging generally occurs in high frequency and low frequency regimes, which would be reflected in the frequency response pattern of sensory ERP (Ciorba et al., 2011; Rigters et al., 2016). Each participant received the auditory stimuli through earphones at a volume level of 70 dB. The duration of each stimulus was 50 ms, with rise and fall times being within 1 ms and the interval between consecutive stimuli being 1 s.

To elicit the selective-attention ERP, we adopted an active auditory oddball task presenting 64 rare random-sequenced target stimuli of 2,000 Hz (1/5 ratio) and 256 monotonic standard auditory stimuli of 750 Hz (4/5 ratio). The stimulus presentation was the same as that adopted to elicit the sensory ERP. The participants were asked to press a response key upon recognition of the target stimuli. The recordings were conducted while the participants kept their eyes closed in a soundless room with regular illumination.

### Preprocessing and Variable Extraction

We tested data for contamination due to muscle and eye movement of the (Fp1, Fp2) prefrontal EEG signals as we did not reject any artifact in the signal processing. First, we checked that none of the EEG data were contaminated by large amounts of artifacts. Specifically, none of the participants contained more than 10% of epochs exceeding 200 μV in maximum amplitude; this value was a common exclusion threshold of each epoch due to serious artifacts (Noh et al., 2006). When applying a stricter voltage threshold of 100 μV, we still found no participants for whom 10% of the epochs exceeded this threshold. Therefore, none of the eye-closed resting-state EEG data were rejected due to artifacts in this study.

Frequency-domain (or spectral-domain) features are typically used in the quantitative analysis of EEG rhythms. To transform an EEG signal from the time domain into the frequency domain, a Fourier transform of the autocorrelation function was employed to provide the power spectral density. In the eye-closed resting EEG, intrinsic oscillation reflective of an idling cortical state becomes dominant, and the dominant peak frequency is usually located in the 4–13 Hz band. Previous reports have commonly revealed that the dominant oscillatory frequencies that appear in the alpha band during normal aging become lower in cognitively disordered patients (Jackson and Snyder, 2008; Jelic and Kowalski, 2009).

Some of the variables used in the resting-state EEG results are explained further. The resting-state EEG markers were derived from a frequency-domain analysis of EEG data measured over 5 min. Concretely, the median frequency measures the average frequency and the peak frequency measures the frequency at the maximum peak, in the dominant intrinsic oscillatory frequency band of 4–13 Hz of the EEG power spectrum. The alpha-to-theta ratio measures the power ratio of alpha rhythms (8–12.99 Hz) to theta rhythms (4–7.99 Hz). The EEG power spectrum was obtained by fast Fourier transform of the EEG signal using a rectangular window. The median frequency was calculated in two steps. Step 1: all spectral power values in the 4–13 Hz frequency domain were summed and divided by 2. Step 2: the frequency at which the cumulative power in the 4–13 Hz frequency domain first, exceeded the value calculated in step 1 was selected. The peak frequency was determined as the frequency at which the power of the EEG spectrum in the 4–13 Hz frequency domain was largest. The absolute power was calculated in the following four frequency regions: delta (0–3.99 Hz), theta (4–7.99 Hz), alpha (8–12.99 Hz), and beta (13–30 Hz). The power data were then logarithmically transformed to fulfill the normal distributional assumptions required for parametric statistical analysis (Choi et al., 2020). The alpha-to-theta ratio was obtained by dividing the alpha power by the theta power, and the frontal asymmetry was obtained by taking the difference between the right and left alpha powers and dividing by their sum.

The ERP markers were derived from event-related potentials extracted by the conventional ensemble averaging method in EEG with stimuli. Sensory ERP variables that are exogenous sensory components represent sensory processes that mainly depend on the stimuli physical parameters and also can be influenced by cognitive processes (Pratt, 2012). The selective attention ERP components measure higher processes of cognitive function, which are related to endogenous cognitive activity (Woodman, 2010). Five variables were considered from the sensory ERP results: The average voltage peak (amplitude), average response time, amplitude deviation, response time deviation, and center-to-edge amplitude difference. Four variables were extracted from the selective-attention ERP results: the number of correct responses, response time, weighted error percentile, and voltage peak difference between the response and background ERPs. Voltage peak is the maximum amplitude of the ERP signal. The response time is the time corresponding to the voltage amplitude peak and is calculated relative to the stimulus onset. All markers were averaged over the left and right signals. The extracted variables are summarized in Table 1.

**Table 1:** EEG/ERP variables considered in this study.

Type	Variable	Notation	Unit	Definition/description	Alteration in dementia patients
Resting-state EEG	Median frequency	MEF	Hz	Frequency at which the cumulative power spectral density between 4 and 13 Hz is divided into two equal amounts (the 50% quantile). Median frequency is obtained by 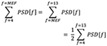 PSD, power spectral density	Median frequency and peak frequency decrease in dementia patients (Garcés et al., 2013; Nina et al., 2014; Babiloni et al., 2018; Rossini et al., 2020)
	Peak frequency	–	Hz	Frequency at which peak power occurs in 4–13 Hz	
	Peak power	–	μV^2^	Maximum PSD amplitude in 4–13 Hz	Shift to lower frequency in peak power in dementia patients (Raicher et al., 2008; Rodriguez et al., 2011)
	Alpha power	Alpha (avg.)	μV^2^	Alpha band (8–12.99 Hz) power averaged over left and right hemispheres	General reduction in alpha band power is an EEG hallmark in AD (Li et al., 2020)
	Theta power	Theta (avg.)	μV^2^	Theta band (4–7.99 Hz) power averaged over left and right hemispheres	Theta power significantly increases in patients with AD dementia. There are significant correlation between relative theta power and multiple neuropsychological measures and total tau proteins (Rodriguez et al., 2011; Vecchio et al., 2011; Musaeus et al., 2018)
	Beta power	Beta (avg.)	μV^2^	Beta band (13–30 Hz) power averaged over left and right hemispheres	Decrement in relative and absolute beta band power was found in dementia patients (Coben et al., 1983; Holschneider and Leuchter, 1995; Christov and Dushanova, 2016)
	Alpha-to-theta ratio	Alpha/theta	–	Ratio of alpha to theta band power Alpha-to-theta ratio = alpha/theta	Lower alpha-to-theta ratio in early and moderate AD patients (Cibils, 2002; Schmidt et al., 2013; Choi et al., 2019)
	Frontal asymmetry	–	–	Asymmetry ratio of alpha band power between right and left hemispheres: *FA* = (*R* − *L*) / (*R* + *L*), *R* (*L*), absolute alpha band power from right (left) hemisphere	Alpha asymmetry is mainly reported in depression-related diseases as greater alpha power in the left frontal region in patients with major depression (Jesulola et al., 2017; Roh et al., 2020)
Sensory ERP	Voltage amplitude peak	Amplitude	μV	Voltage peak of ERP responses averaged over different frequencies	Sensory ERP components were found to be relatively low in sensitivity to detect changes in dementia (Hirata et al., 2000; Olichney et al., 2011)
	Response time	–	ms	Mean time delay between stimulus and response (i.e., voltage peak) averaged over different frequencies	Delayed response across different auditory and visual oddball tasks in dementia patients (Cecchi et al., 2015; Gu et al., 2018)
	Voltage amplitude deviation	Amplitude (deviation)	μV	Standard deviation between voltage peaks over different frequencies	–
	Response time deviation	Response time (deviation)	ms	Standard deviation between response times over different frequencies	–
	Center-to-edge amplitude difference	Amplitude (edge-center ratio)	–	Mean voltage peaks at 500, 750, 1,500, and 2,000 Hz minus mean voltage peaks at 125 and 4,000 Hz divided by their sum	–
Selective-attention ERP	Number of correct responses	# of correct	–	Number of correct responses for target stimulus (2,000 Hz tone) distinguished from background stimulus (750 Hz tone)	Reduced accuracy in ERP-related tasks in dementia patients (Mathalon et al., 2003; Cecchi et al., 2015; Gu et al., 2018)
	Response time	Resp. Time	s	Time between auditory stimulation and voltage peak of EEG voltage oscillations	Response time to evoked auditory stimuli increases in dementia patients (Yener and Başar, 2010; Gu et al., 2018)
	Weighted error percentile	wER	–	wER = (no. errors + 4 × (64 − no. correct recognitions)/(256 + 64 × 4) No. of target (background) stimuli = 64 (256)	–
	Amplitude difference between response and background ERP	Amp (resp)– Amp (bg)	μV	Difference in voltage peaks of EEG oscillations between target and background stimuli	Patients with AD dementia showed lower amplitude for ERP features (Vecchio and Määttä, 2011; Cecchi et al., 2015)

*Eight variables from resting-state EEG, five from sensory ERP, and four from selective attention ERP. EEG, electroencephalography; ERP, event-related potential*.

### Statistical Analysis

The significant level for all statistical tests is set to α = 0.05. The demographic and neuropsychological characteristics were summarized as the means and standard deviations (SDs) or medians and ranges (from minimum to maximum values) for continuous variables, and as the frequencies and proportions for categorical variables for the dementia and non-dementia groups. Either an independent two-sample *t*-test or a Mann-Whitney-Wilcoxon rank-sum test was performed after checking the normality of each group of data based on the Shapiro-Wilk test to assess the differences in the continuous variables across dementia and non-dementia individuals. The chi-squared (χ^2^) test or the Fisher’s exact test was used to check the independence of the categorical variables from the dementia status. The association between the MMSE score and each EEG/ERP variable was evaluated using the Pearson’s correlation coefficient ($\hat{\rho }$) and slope of each EEG measurement ($\hat{\beta }$) obtained from univariate linear regression analysis.

The distribution of every EEG/ERP variable for the dementia and non-dementia individuals was obtained using kernel density estimation to visualize the natural differences in both groups for illustrative purposes. Univariate and multiple logistic regression analyses were conducted to estimate the unadjusted or adjusted odds ratios for dementia in each EEG/ERP variable to assess the associations between the EEG/ERP variables and dementia prevalence. In the multiple logistic regression analysis, sex, age, education level, and GDS score were used as covariates. The underlying diseases of the participants described in Table 2 were not considered as covariates due to the small sample size. Furthermore, the MMSE score was included as an additional covariate in the regression model to identify the independent association of the EEG/ERP variables for dementia.

**Table 2:** Demographic information and neuropsychological test results of dementia and non-dementia subjects.

	Total (*n* = 122)	No (*n* = 87)	Yes (*n* = 35)	Test Statistic
**Age [years]**
Mean (SD)	71.0 (±11.9)	68.2 (±11.2)	78.1 (±10.7)	
Median [range]	73.9 [42.3–95.9]	68.7 [48.3–90.6]	78.6 [42.3–95.9]	W = 777.0, *p* = 0.0000
**Sex**
Male	30 (25%)	22 (25%)	8 (23%)
Female	92 (75%)	65 (75%)	27 (77%)	$\chi_{\left(df=1\right)}^{2}$ = 0.0, *p* = 9.605E-1
**Education level [year]**
Mean (SD)	6.0 (±5.0)	7.1 (±5.0)	3.4 (±3.9)	W = 2,148.5, *p* = 0.0003
Median [range]	6.0 [0.0–18.0]	6.0 [0.0–18.0]	0.0 [0.0–12.0]
**Systolic BP [mmHg]**
Mean (SD)	125.6 (±16.4)	125.1 (±15.5)	126.9 (±18.6)	*t*120.0 = –0.6, *p* = 0.5716
Median [range]	123.5 [80.0–170.0]	123.0 [80.0–170.0]	130.0 [98.0–169.0]
**Diastolic BP [mmHg]**
Mean (SD)	73.5 (±11.7)	74.3 (±12.0)	71.4 (±10.8)	*t*120.0 = 1.3, *p* = 0.2091
Median [range]	70.0 [41.0–100.0]	73.0 [41.0–100.0]	70.0 [45.0–95.0]
**MMSE score**
Mean (SD)	23.2 (±5.7)	25.3 (±4.6)	18.0 (±5.0)	W = 2630.0, *p* < 1E-6
Median [range]	25.0 [5.0–30.0]	27.0- [12.0–30.0]	18.0 [5.0–28.0]
**GDS score**
Mean (SD)	12.2 (±6.5)	10.7 (±6.0)	16.0 (±6.1)	W = 810.5, *p* = 0.0001
Median [range]	11.0 [1.0–28.0]	9.0 [1.0–24.0]	16.0 [2.0–28.0]
**Diabetes**
No	101 (83%)	73 (84%)	28 (80%)	$\chi_{\left(df=1\right)}^{2}$ = 0.1, *p* = 0.8010
Yes	21 (17%)	14 (16%)	7 (20%)
**Hypertension**
No	60 (49%)	46 (53%)	14 (40%)	$\chi_{\left(df=1\right)}^{2}$ = 1.2, *p* = 0.2774
Yes	62 (51%)	41 (47%)	21 (60%)
**Hyperlipidemia**
No	105 (86%)	73 (84%)	32 (91%)	FE-test, *p* = 0.3900
Yes	17 (14%)	14 (16%)	3 (9%)
**Thyroid disease**
No	115 (94%)	81 (93%)	34 (97%)	FE-test, *p* = 0.6718
Yes	7 (6%)	6 (7%)	1 (3%)
**Mental disorder**
No	93 (76%)	81 (93%)	12 (34%)	$\chi_{\left(df=1\right)}^{2}$ = 44.5, *p* < 1E-6
Yes	29 (24%)	6 (7%)	23 (66%)
**Nervous system disease**
No	111 (91%)	79 (91%)	32 (91%)	FE-test, *p* = 1.0000
Yes	11 (9%)	8 (9%)	3 (9%)
**Circulatory disease**
No	118 (97%)	84 (97%)	34 (97%)	FE-test, *p* = 1.0000
Yes	4 (3%)	3 (3%)	1 (3%)

*The values represent the mean (±SD), median and range (minimum–maximum) for the continuous variables and N (%) for the categorical variables. The test statistics and *p*-values for the continuous variables were obtained from an independent two sample *t*-test (*t* value with degree of freedom, df) or a Mann-Whitney-Wilcoxon rank sum test (W) after checking the normality of each group of data based on the Shapiro-Wilk test. For the categorical variables, the *p*-values were derived from the chi-squared test statistics. FE-test: Fisher’s exact test*.

Dementia prediction models were developed based on all EEG/ERP and demographic variables (age, sex, and education level) that are directly associated with cognitive status. The MMSE score was also used as a single predictor to compare the performances of the models using EEG/ERP features or to investigate the improvement of the predictive models using EEG/ERP features in combination with the MMSE score. All continuous predictors were standardized to a mean of 0 and SD of 1 for data preprocessing. For the model comparisons, we generated 12 datasets based on combination of the variable groups: MMSE score, demographics, resting-state EEG, sensory ERP, and selective attention ERP. The interaction terms between sex and other variables were included as predictors in each candidate model containing demographic features.

In total, 122 participants were randomly split, with 80% being in the training set (*n* = 98) and 20% in the test set (*n* = 24). The dementia cases in both the training and test sets were distributed proportionally to the total sample size. Before assigning data to the training and test sets, the total dataset was stratified by the dementia status. Consequently, 20% of the data were randomly selected according to each stratum, and then the selected data from both strata were merged into the test dataset. The rest 80% of the data of both strata were merged into the training set. We trained several learning algorithms using a five-fold cross-validation approach, for which the training dataset was again stratified according to the dementia status; subsequently, the randomly generated fold identifiers were given to each stratified group. The learning algorithms employed in this study included binary logistic regression with stepwise variable selection based on Akaike information criteria; penalized logistic regression including ridge, elastic net, and least absolute shrinkage selection operator (Friedman et al., 2020); random forest algorithm (Wright et al., 2020); and extreme gradient boosting (Chen et al., 2020). The model performance was evaluated using the AUROC and binomial deviance. The optimal model (with the highest AUROC and lowest binomial deviance) was selected within each combination of learning algorithms and 12 datasets, and its prediction power was evaluated with the test set. All statistical analyses and predictive model development were conducted using the statistical software R (version 4.0.2, released 2019-06-22; R Core Team, 2020).

## Results

“Subject Characteristics” section describes the basic characteristics of the participants with regard to their demographics, neuropsychological information, and comorbidities. “Correlation Between MMSE Score and EEG Measures” section demonstrates the correlation between the EEG/ERP variables and conventional MMSE scores for screening dementia using linear regression analysis. “Densities of EEG/ERP Variables Between Dementia and Non-dementia Subjects” section reports the distribution of each EEG/ERP variable by its density in the dementia and non-dementia groups. “Relation Between EEG/ERP Variables and Dementia” section clarifies the relations between the EEG/ERP variables and dementia, using the estimated odds ratios in the unadjusted and the two adjusted models based on logistic regression. Finally, “Prediction Models for Dementia” section provides an evaluation of the various dementia prediction models based on the EEG/ERP variables, MMSE scores, and demographic data.

### Subject Characteristics

The overall demographic information, neuropsychological characteristics, and comorbidities of the 122 persons enrolled in this study are listed in Table 2. Among the participants, 87 were non-dementia individuals, and the remaining 35 were confirmed dementia patients. Further, 25% of the participants were male and the remaining 75% were female. The ages of the dementia and non-dementia groups were 78.1 ± 10.7 and 68.2 ± 11.2 years (mean ± standard deviation), respectively, and their education levels were 3.4 ± 3.9 and 7.1 ± 5.0 years (p < 0.05), respectively. The MMSE score was 18.0 ± 5.0 for the dementia patients and 25.3 ± 4.6 for the non-dementia individuals and ranged from 5.0–30.0 (p < 0.05). Thus, the dementia patients exhibited lower MMSE scores and education levels and higher mean ages than the non-dementia individuals (Pedraza et al., 2013; Qin et al., 2020). Moreover, the GDS score was higher in the dementia individuals (16.0 ± 6.1, mean ± standard deviation) than in the non-dementia subjects (10.8 ± 6.2). The physiological and psychological information, such as blood pressure, diabetes, hypertension, and mental disorders, showed no statistically significant differences.

### Correlation Between MMSE Score and EEG Measures

**Figure 2:**
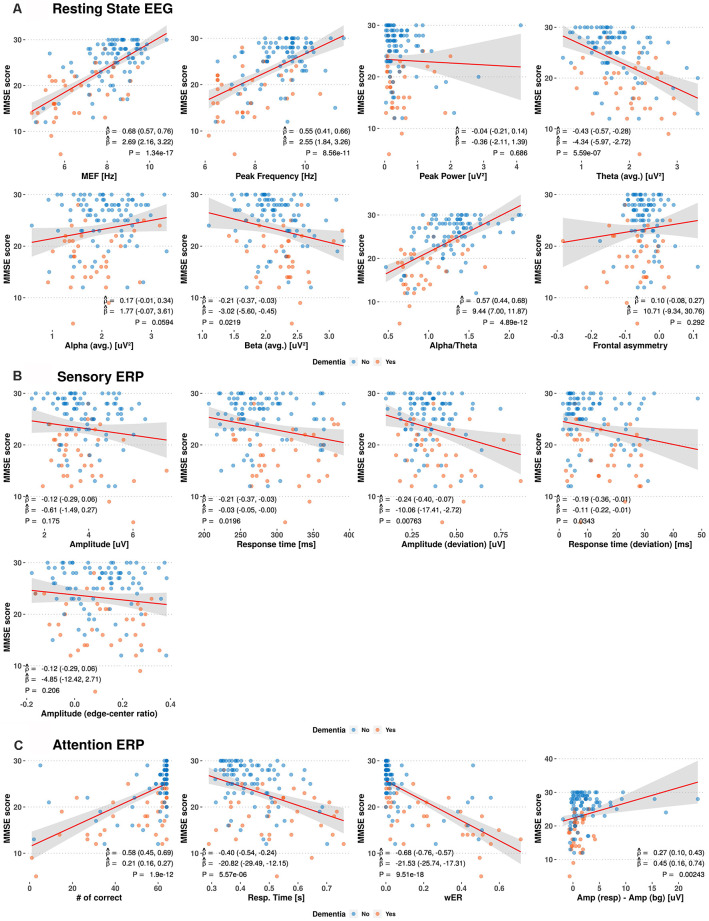
Scatterplots between Mini-Mental Status Examination (MMSE) scores and electroencephalography (EEG)/event-related potential (ERP) variables. The red and blue circles indicate the dementia and non-dementia subjects, respectively. The red line and shaded area show the estimated regression curves and 95% confidence intervals derived from univariate regression analysis. The estimated Pearson correlation coefficient ($\hat{\rho }$), regression coefficient ($\hat{\beta }$), and *p*-value (P) for each EEG/ERP variable are shown with their 95% confidence intervals (MEF, median frequency; wER, weighted error percentile).

We investigated the relations between the MMSE score and prospective EEG/ERP variables from the resting-state EEG, sensory ERP, and selective-attention ERP using linear regression models and the Pearson correlation coefficients, obtaining the results shown in Figure 2. Weak to moderate linear correlations are observed between the MMSE score and EEG/ERP variables.

Among the resting-state EEG variables, the median frequency, peak frequency, and alpha-to-theta ratio show positive moderate linear correlations with the MMSE score, with average Pearson correlation coefficient ($\hat{\rho }$) of 0.55–0.68 and average regression coefficients ($\hat{\beta }$) of 2.55–10.29. The theta power shows a negative linear correlation with the MMSE score, with $\hat{\rho }$ = −0.43 and $\hat{\beta }$ = −4.34. Individual variables from the sensory ERP show weak negative correlations with the MMSE scores, with $\hat{\rho }$ from −0.12 to −0.24. For the selective-attention ERP variables, the MMSE scores show moderate linear correlations with the most variables, including positive correlations with the number of correct responses and amplitude difference between responses, with $\hat{\rho }$ = 0.58 and $\hat{\rho }$ = 0.27, respectively, and negative correlations with the response time and weighted error percentile, with $\hat{\rho }$ ranging from −0.40 to −0.68.

### Densities of EEG/ERP Variables Between Dementia and Non-dementia Subjects

**Figure 3:**
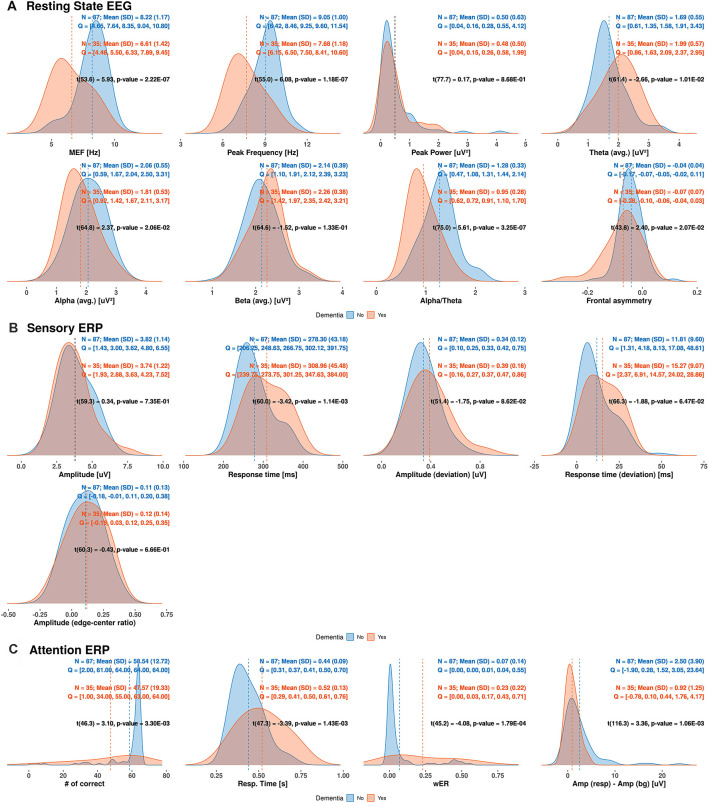
Estimated densities of EEG/ERP variables in dementia and non-dementia subjects. Q indicates values divided into four quantiles (MEF, median frequency; wER, weighted error percentile; SD, standard deviation).

We determined the distribution of each EEG/ERP variable based on its density in the dementia and non-dementia groups, obtaining the results shown in Figure 3. Overlapping distributions are observed for some variables obtained from the resting-state EEG and sensory ERP results. However, the variables exhibiting moderate correlations with the MMSE score (reported in “Correlation Between MMSE Score and EEG Measures” section) consistently show significant differences between the dementia and non-dementia groups. Specifically, the median frequency, peak frequency, alpha-to-theta ratio, and theta power from the resting-state EEG results; average response time from the sensory ERP results; and all selective-attention ERP variables exhibit significant differences between the dementia and non-dementia groups. Overall, the observed differences in the distributions of the EEG/ERP variables reflect the different cognitive statuses of the dementia and non-dementia groups.

### Relation Between EEG/ERP Variables and Dementia

**Figure 4:**
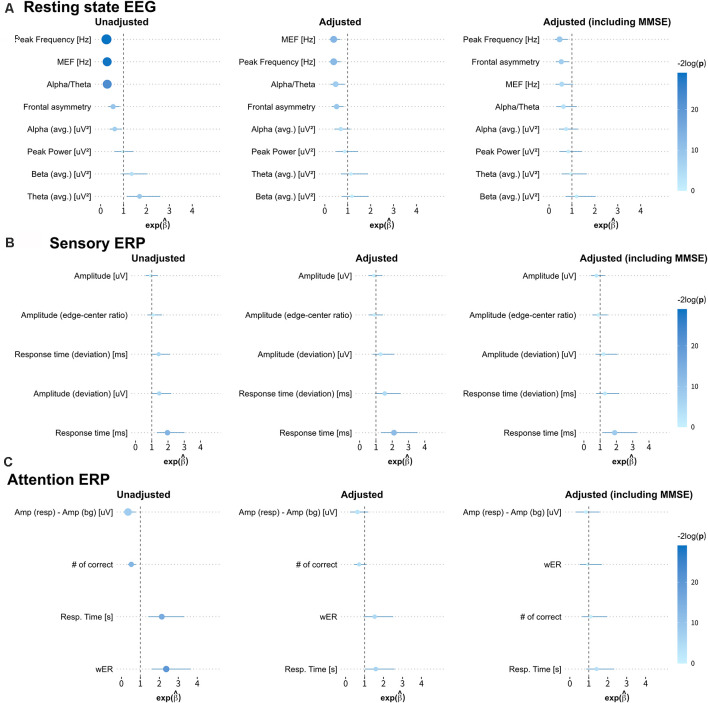
Estimated odds ratios and 95% confidence intervals derived from three logistic regression models. The first, second, and third columns show the results from the unadjusted univariate logistic regression model; the model adjusted for sex, age, education level, and geriatric depression scale (GDS) score; and the model adjusted for all these covariates and the MMSE score, respectively. The size of each circle indicates the magnitude of the estimated odds ratio, and the line across the circle represents its 95% confidence interval. The color map represents the magnitude of the log-transformed *p*-values (−2log p) for the odds ratios (MEF, median frequency; wER, weighted error percentile). The quantitative results are summarized in Appendix Table A1.

We obtained the forest plots shown in Figure 4 for the estimated odds ratios and the 95% confidence intervals of the EEG/ERP variables for predicting dementia. Three logistic regression models were considered, namely, the unadjusted model (first model); the first model adjusted for sex, age, education level, and GDS score (second model); and the second model also adjusted for the MMSE score (third model).

In the first model, most variables from the resting-state EEG and selective-attention ERP reflect the risk of dementia, with odds ratios and 95% confidence intervals, significantly different from 1. Specifically, small peak frequency, median frequency, alpha-to-theta ratio, frontal asymmetry, and large theta band power in the resting-state EEG results indicate increased risk of dementia with mean odds ratios of 0.255, 0.285, 0.289, 0.546, and 1.699 (*p*-values from 1.09E-2 to 5.58E-7), respectively. Similarly, all variables from the selective-attention ERP contribute with mean odds ratios from 0.349–0.521 and from 2.130–2.364 (*p*-values from 1.96E-2 to 4.31E-5). In addition, the delayed average response time between the left and right hemispheres in sensory ERP also indicates increased risk of dementia with a mean odds ratio of 1.967 (*p*-value of 1.25E-3). The detailed odds ratios and *p*-values are presented in Appendix Table A1.

In the second model, only the median frequency, peak frequency, alpha-to-theta ratio, and frontal asymmetry in the resting-state EEG results and the average response time in the sensory ERP results are effective to identify dementia after adjustment, with odds ratios and 95% confidence intervals different from 1. Notably, the bounds of the 95% confidence intervals of these variables in the second model are wider than those in the first model, but they still reflect the risk factors of dementia with *p* < 0.05. Hence, the median frequency, peak frequency, alpha-to-theta ratio, frontal asymmetry and average response time tend to be independent from the demographic risk factors and may represent risk factors of dementia.

In the third model, most variables correlated with the MMSE score no longer represent risk factors, and only a few variables, including the peak frequency and frontal asymmetry in the resting-state EEG results and average response time in the sensory ERP results enable identification of dementia. Interestingly, frontal asymmetry shows no correlation with the MMSE score, but it represents a considerable risk factor for dementia after adjustment for demographic covariates and the MMSE score.

Variables such as the median frequency, peak frequency, and alpha-to-theta ratio in the resting-state EEG results present moderate to strong correlations with the MMSE score (reported in “Correlation Between MMSE Score and EEG Measures” section) and provide consistent odds ratio values for classifying dementia. On the other hand, the frontal asymmetry in the resting-state EEG results and the average response times from the left and right hemispheres in the sensory ERP results show no or weak correlations with the MMSE score. Nevertheless, they exhibit valid odd ratios for classifying dementia, suggesting that they could be candidate biomarkers for dementia screening independently from the MMSE score.

### Prediction Models for Dementia

We categorized the prediction model results into five groups (Table 3). The first group contained univariate analysis of MMSE, multivariate analysis of individual sets of MMSE plus demographic information, resting-state EEG, sensory ERP, and selective-attention ERP. The logistic regression model using the ordinary least squares approach for parameter estimation predicted dementia using only the MMSE score, achieving a 0.803 AUROC and 23.845 deviance. Adding demographic information to the MMSE score did not improve the accuracy. In fact, the prediction model based on logistic regression plus elastic net using the MMSE score and demographic information provided equal AUROCs of 0.803 with deviances of 25.234. In this first group, the predictor based on selective-attention ERP variables yielded the highest AUROC of 0.891 and lowest deviance of 20.363 using logistic regression. In addition, the resting-state EEG variables enabled higher accuracy than the MMSE score or the MMSE score combined with demographic information.

**Table 3:** Evaluation results of prediction models according to type of data and classification model.

	Logistic regression (Ordinary least square)		Logistic regression + Elastic net		Random forest		Extreme gradient boosting	
	AUROC	Deviance	AUROC	Deviance	AUROC	Deviance	AUROC	Deviance
MMSE	**0.803**	**23.845**	-	-	-	-	-	-
DM + MMSE	0.664	31.380	0.803	25.234	0.748	26.200	0.752	26.995
RSEEG	**0.824**	**23.037**	0.824	22.183	0.773	23.843	0.807	23.929
sensERP	0.697	30.332	0.647	28.832	0.605	28.465	0.500	28.979
attERP	**0.891**	**20.363**	0.882	21.608	0.857	20.679	0.882	24.134
RSEEG + sensERP + attERP	0.739	42.722	0.849	21.146	0.832	22.569	0.849	21.193
DM + RSEEG + sensERP + attERP	0.571	73.514	0.832	21.439	0.832	21.954	0.832	21.295
MMSE + RSEEG	0.798	25.300	0.849	22.141	0.807	21.581	0.849	22.123
MMSE + sensERP	0.807	25.795	0.798	23.338	0.790	22.598	0.756	29.949
MMSE + attERP	0.803	23.845	0.849	23.064	0.798	24.330	0.739	26.390
MMSE + RSEEG + sensERP + attERP	0.782	40.182	0.866	20.855	0.866	20.875	0.866	20.986
DM + MMSE + RSEEG + sensERP + attERP	0.605	86.032	0.849	22.048	0.866	21.140	0.874	21.150
Significant-variables	0.874	20.628	**0.891**	**19.397**	0.798	22.908	0.874	20.461

*The MMSE score, demographic information (i.e., sex, age, education level, interaction between sexes, and other variables), resting-state EEG (eight variables), sensory ERP (five variables), and selective-attention ERP (four variables) were the data sources. For the models with demographic variables, the terms corresponding to the interaction between sex and other variables were included in each model. DM, Demographic information; RSEEG, resting state EEG; sensERP, sensory ERP; attERP, selective-attention ERP. For models with demographic variables, the terms corresponding to the interaction between sex and other variables are included in each model.“Significant-variables” models contain MEF, peak frequency, alpha/theta, frontal asymmetry from resting state EEG, response time from sensory ERP, and number of correct responses, response time and weighted error percentile from attention ERP. The bold fonts indicate outstanding prediction accuracies*.

The second group combined resting-state EEG, sensory ERP, and selective-attention ERP before and after adding demographic information. Loosely speaking, these prediction models failed to improve accuracy compared with the models from the EEG/ERP variables in the first group.

The third group combined the MMSE score with different EEG/ERP variables. Combining the MMSE score with resting-state EEG or sensory ERP provided better prediction accuracy than using the EEG variables from the single groups or the MMSE score alone, reaching an AUROC of 0.849 and a deviance of 22.141 when using logistic regression and elastic net regularization. In comparison with selective-attention ERP alone, combining the MMSE score with selective-attention ERP or with all three EEG/ERP variables did not increase the prediction accuracy.

The fourth group combined demographic information with the MMSE score, resting-state EEG, sensory ERP, and selective-attention ERP. This group provided a lower AUROC with higher deviance than the third group, in which demographic information was neglected.

Finally, the fifth group (“significant-variables” model) contained eight most likely potential markers among all the variables, including MEF, peak frequency, alpha-theta, and frontal asymmetry from resting state EEG, response time from sensory ERP, and number of correct responses, response time, and weighted error percentile from selective-attention ERP. These variables were shown to have high correlations with MMSE score (“Correlation Between MMSE Score and EEG Measures” section), less overlapping in their distribution between dementia and non-dementia groups (“Densities of EEG/ERP Variables Between Dementia and Non-dementia Subjects” section), and indicated as risk factors of dementia after adjusting for covariates (“Relation Between EEG/ERP Variables and Dementia” section). This combination provided a similar AUC of 0.891 but lower deviance of 19.397 using logistic regression with elastic net in comparison with selective-attention ERP cluster (deviance 20.363) using logistic regression with ordinary least square. In this “significant-variables” model based on the elastic net, the accuracy went up to 92.7%.

The prediction model results show that the groups of resting-state EEG and selective-attention ERP variables predict dementia better than the MMSE score. In addition, the EEG/ERP variables combined with the MMSE score further improve dementia prediction, except for selective-attention ERP, whereas adding demographic information to either the EEG/ERP variables or MMSE score does not improve the prediction accuracy. The ineffectiveness of demographic information may be due to the diversity of the participants and the small sample size. The evaluation results of the prediction models are summarized in Table 3.

## Discussion

In this study, spontaneous resting state EEG, sensory ERP and selective-attention ERP were used as three methods to obtain the important brain oscillations (Başar et al., 2016). Both EEG and ERP variables have been investigated as potential biomarkers to detect MCI and its progression to AD dementia, as well as to directly detect AD dementia (Herrmann and Demiralp, 2005; Uhlhaas and Singer, 2006; Jackson and Snyder, 2008). In resting-state EEG, frequency components shift from high-frequency bands (i.e., alpha and beta) to lower frequency bands (i.e., delta and theta), and the alterations develop gradually according to the disease severity (Jelles et al., 2008; Smailovic and Jelic, 2019). Similarly, the peak frequency, median frequency, and alpha-to-theta ratio in dementia patients drift towards lower frequencies compared with non-dementia individuals (Raicher et al., 2008; Dauwels et al., 2010; Schmidt et al., 2013). In ERP, amplitude reduction and increased latency have been reported (Başar et al., 2010), as well as reduced accuracy and increased response time in a target detection task (Cecchi et al., 2015) in dementia patients. Our findings are consistent with the results of these studies.

The MMSE has been used widely in clinical practice as an effective and sensitive test to detect and screen cognitive impairment and dementia (Benson et al., 2005; Arevalo-Rodriguez et al., 2015). The MMSE enables dementia detection with 92% accuracy, 78%–84% sensitivity, and 87%–91% specificity (cutoff value of 23/24) (Tsoi et al., 2015). However, the MMSE has bias according to the socio-educational backgrounds of participants, practice effect, and low sensitivity in the early stage of cognitive decline (Scazufca et al., 2009; Duff et al., 2012; Carnero-Pardo, 2014). These disadvantages can be overcome while enhancing the diagnostic accuracy by combining the MMSE score with EEG/ERP data.

Selective-attention ERP examines the cognitive performance using auditory oddball paradigm, which elicits P300 in response to the target intonations through the use of prompt button pushing. This motoric response can cause a distinct movement-related potential, which has been reported to interfere with the topography of P300 and alter its amplitude in comparison with the silent-count task (Salisbury et al., 2001; van Vliet et al., 2014; Kim et al., 2020). Despite these reported influences on P300 with button-pushing behavior, for old participants with as many as 64 deviant stimuli, the button-press was an optimal task touse the counts of correct and erroneous responses as the two salient variables in evaluating cognitive performance.

Selective-attention ERP variables include the number of correct responses, response time, weighted error percentile, and amplitude difference between deviant and background stimuli. Selective attention ERP has been shown to provide the highest AUROC values, while demonstrating the best dementia predictor among all the possible combinations of dementia risk factors. Selective-attention ERP or attention components of P300 have been studied as indicators for cognitive processing. Selective-attention ERP endogenous components reflect the ability of cognitively processing the stimulus based on the levels of attention and arousal (Polich and Kok, 1995). A prolonged P300 response time implies that more time is required to process information, which represents an index of abnormal cognition ability (Williams et al., 1991; van Deursen et al., 2009). P300 amplitude reduction in dementia patients shows that lower attentional resources were devoted to the task performance (van Deursen et al., 2009; Hedges et al., 2016). Furthermore, decreasing number of correct answers and increasing weighted error percentile in the dementia group as compared to those in the normal group indicate a reduction in attentional maintenance and action control ability during cognitive processing throughout the task (Vecchio and Määttä, 2011). All changes in selective-attention ERP variables indicate a decrease in intrinsic brain activation to the responses in demented patients. Selective-attention ERP provides a sensitive and reliable measure for the early detection of cognitive impairment related to AD (Cecchi et al., 2015; Gu et al., 2018). Our findings upheld the literature associated with using attention ERP for detecting dementia.

As indicated by the significant odds ratios before and after adjusting for sex, age, education level, and GDS score, the EEG/ERP variables show high correlations with the MMSE score and indicate dementia risk factors. Furthermore, variables with low correlations with the MMSE score (e.g., frontal asymmetry in resting-state EEG) may be suitable for classifying dementia independently from the MMSE score, as indicated by the significant odds ratios that are obtained after adjusting for the covariates plus the MMSE score. Frontal asymmetry has been used as an indicator of depression due to the hyperactivity of the right prefrontal lobe and the withdrawal behavior to aversive stimuli (Thibodeau et al., 2006; Jesulola et al., 2015). However, to the best of our knowledge, frontal asymmetry has not been reported as a candidate indicator of dementia. Thus, our findings establish a new direction for research on dementia by considering frontal alpha asymmetry.

Considering dementia and its relation to depression, half of the patients with late-onset depression may exhibit cognitive impairment, and the prevalence of depression in dementia patients is between 9% and 68% (Muliyala and Varghese, 2010). Asymmetry in frontal cortex activity reflected in EEG signals has been described as a potential discriminator for depression, such that frontal alpha asymmetry has been found to be significantly higher in depressed subjects than healthy controls (Gollan et al., 2014; Adolph and Margraf, 2017; Brzezicka et al., 2017); however, contradicting results have also been reported (van der Vinne et al., 2017; Kaiser et al., 2018). Our results may suggest that the frontal alpha asymmetry as one of the potential EEG variables for dementia detection.

We derived prediction models using different combinations of EEG/ERP variables, MMSE scores, and demographic data. Selective-attention ERP variables and resting-state EEG variables produced more accurate predictions than MMSE scores or MMSE scores combined with demographic information. Hence, these variables may be representative in the identification of cognitive changes due to dementia. In contrast, adding demographic information tended to decrease the accuracy compared to the cases in which demographic information was neglected. Hence, demographic information may undermine predictive modeling of dementia.

The variable selection in the prediction model based on the statistical test often leads to serious bias in maximizing the performance of the predictive model, as explained by Lo et al. (2015). To overcome this limitation, we adopted penalized regression approaches that performed the variable selection continuously. In our case, a model with the variables that showed highest statistical significances resulted in best accuracy among various prediction models (Table 3). In particular, the model exhibiting the highest AUROC (0.891) and lowest deviance (19.397) employed the eight most significant-variables in the logistic regression approach with elastic net regularization, followed by the selective-attention ERP variables in a logistic regression model via the ordinary least squares method. It implies that a prediction model with only few EEG/ERP variables that showed high statistical significance can be used for effective screening of dementia, which would lead to the cost effective utility of “prefrontal EEG” in clinics.

Overall, the logistic regression model with elastic net regularization tended to perform better than the random forest or extreme gradient boosting approach in terms of AUROC and deviance from individual EEG/ERP variables with or without MMSE. Again, adding demographic information to this model reduced the predictive performance. The adverse effects of demographics may be due to the diversity of participants considered in this study regarding aspects such as age, sex, education level, GDS score, and the underlying disease causing dementia.

Some limitations of this study remain to be addressed. The dementia patients in this study were registered in the Korean National Health Insurance Service, and we were not able to obtain further medical records of the patients, such as imaging data, to identify the underlying causes and statuses of dementia. Therefore, hidden comorbidities inducing diversity of EEG/ERP features may have affected our results. In addition, our findings cannot be generalized due to the small sample size (122 participants) and discrepancies in age and education level among groups. Even though we attempted to remove confounding effects by adjusting for age, sex, education level and depression level, the prediction models could increase clinical usability if the data had no such discrepancies in other risk factors between dementia patients and normal controls. Finally, we could not examine the exposures or suspected risk factors over time. Thus, a prospective or case-control study with a larger and more representative sample is still required to clinically validate the diagnostic value of the EEG/ERP variables considered in our study.

## Conclusion

Prefrontal EEG variables, which are related to EEG slowing, left–right asymmetry in the resting state, and sensory and selective-attention ERPs, have been correlated with the MMSE score. Logistic regression for dementia prediction shows that most of the selected variables remain significant after adjustment for GDS and demographic risk factors of dementia, such as age, education level, and sex. In contrast, when the model is adjusted for the MMSE score and demographic covariates, these prefrontal EEG variables become non-significant, except for the frontal asymmetry among the activity in the left and right hemispheres, peak frequency in resting-state EEG, and the response time in sensory ERP. The other variables have no or minimal correlations with the MMSE score after such adjustment. From multivariate regression models with five-fold cross-validation, we found that the prefrontal EEG variables outperform the MMSE score in dementia prediction. In particular, the prediction accuracy was the highest when using the eight variables that showed highest statistical significances among tested EEG/ERP variables. Adding demographic information fails to improve the prediction accuracy. Overall, the slowing and asymmetry of prefrontal EEG activity seem promising for dementia screening, and can be used in combination with the MMSE score or function as its alternative. In a future study, the clinical usability of few-channel EEG can be improved by recruiting more participants with balanced demographic risk factors among patient and control groups and by including preceding stages of dementia such as MCI; screening MCI patients effectively allows early medical intervention that can prevent or deter the progression to dementia.

## Data Availability Statement

The original contributions presented in the study are included in the article, further inquiries can be directed to the corresponding author.

## Ethics Statement

The studies involving human participants were reviewed and approved by Declaration of Helsinki. The patients/participants provided their written informed consent to participate in this study.

## Author Contributions

DD led the manuscript preparation. BK analyzed the data and wrote the manuscript. JC extracted relevant EEG variables and controlled the EEG data quality. MO analyzed the data. WC designed and led the Brain Aging Map Project. KK worked to obtain the institutional review board approval. JK designed the study and wrote the manuscript. All authors commented on and approved the contents of the manuscript. All authors contributed to the article and approved the submitted version.

## Conflict of Interest

The authors declare that the research was conducted in the absence of any commercial or financial relationships that could be construed as a potential conflict of interest.

## Figures and Tables

**Table A1:** Estimated odds ratios and 95% confidence intervals derived from three logistic regression models.

		Unadjusted		Adjusted		Adjusted (including MMSE)	
		OR	Wald Z	OR	Wald Z	OR	Wald Z
		(95% CI)	(p-value)	(95% CI)	(p-value)	(95% CI)	(p-value)
**Resting state EEG**	Alpha (avg.) [μV^2^]	0.615 (0.40, 0.93)	−2.26 (2.39E−02)	0.693 (0.42, 1.12)	−1.47 (1.41E−01)	0.747 (0.43, 1.26)	−1.08 (2.80E−01)
	Alpha/Theta	0.289 (0.16, 0.49)	−4.39 (1.14E−05)	0.474 (0.24, 0.87)	−2.33 (1.99E−02)	0.630 (0.32, 1.21)	−1.36 (1.75E−01)
	Beta (avg.) [μV^2^]	1.355 (0.91, 2.05)	1.49 (1.37E−01)	1.186 (0.74, 1.92)	0.71 (4.76E−01)	1.204 (0.72, 2.04)	0.71 (4.78E−01)
	Frontal asymmetry	0.546 (0.34, 0.83)	−2.68 (7.47E−03)	0.516 (0.30, 0.82)	−2.58 (9.75E−03)	0.535 (0.30, 0.87)	−2.29 (2.21E−02)
	Peak frequency [Hz]	0.255 (0.14, 0.42)	−5.01 (5.58E−07)	0.387 (0.20, 0.69)	−3.07 (2.11E−03)	0.460 (0.24, 0.83)	−2.50 (1.26E−02)
	MEF [Hz]	0.285 (0.17, 0.45)	−4.97 (6.64E−07)	0.387 (0.21, 0.68)	−3.19 (1.40E−03)	0.555 (0.28, 1.06)	−1.76 (7.79E−02)
	Peak power [μV^2^]	0.969 (0.61, 1.42)	−0.15 (8.79E−01)	0.868 (0.48, 1.45)	−0.51 (6.13E−01)	0.842 (0.46, 1.43)	−0.60 (5.52E−01)
	Theta (avg.) [μV^2^]	1.699 (1.14, 2.60)	2.54 (1.09E−02)	1.143 (0.70, 1.89)	0.53 (5.94E−01)	0.952 (0.55, 1.65)	−0.18 (8.60E−01)
**Sensory ERP**	Amplitude (deviation) [μV]	1.459 (0.99, 2.19)	1.88 (5.95E−02)	1.282 (0.80, 2.12)	1.01 (3.13E−01)	1.213 (0.71, 2.07)	0.72 (4.72E−01)
	Amplitude (edge−center ratio)	1.094 (0.74, 1.63)	0.45 (6.56E−01)	0.903 (0.57, 1.43)	−0.44 (6.61E−01)	0.884 (0.53, 1.47)	−0.48 (6.33E−01)
	Amplitude [μV]	0.931 (0.62, 1.38)	−0.35 (7.24E−01)	0.875 (0.54, 1.40)	−0.56 (5.78E−01)	0.768 (0.44, 1.30)	−0.97 (3.32E−01)
	Response time (deviation) [ms]	1.424 (0.97, 2.11)	1.79 (7.32E−02)	1.538 (0.95, 2.51)	1.75 (7.96E−02)	1.292 (0.76, 2.17)	0.96 (3.35E−01)
	Response time [ms]	1.967 (1.32, 3.01)	3.23 (1.25E−03)	2.109 (1.31, 3.56)	2.96 (3.09E−03)	1.892 (1.15, 3.26)	2.42 (1.55E−02)
**Attention ERP**	# of correct	0.521 (0.34, 0.77)	−3.18 (1.46E−03)	0.711 (0.45, 1.10)	−1.54 (1.24E−01)	1.106 (0.65, 1.97)	0.36 (7.19E−01)
	Amp (resp) − Amp (bg) [μV]	0.349 (0.13, 0.75)	−2.33 (1.96E−02)	0.626 (0.23, 1.20)	−1.13 (2.59E−01)	0.844 (0.32, 1.57)	−0.43 (6.70E−01)
	Resp. Time [s]	2.130 (1.42, 3.32)	3.53 (4.21E−04)	1.593 (1.01, 2.59)	1.96 (5.04E−02)	1.408 (0.86, 2.34)	1.36 (1.74E−01)
	wER	2.364 (1.59, 3.66)	4.09 (4.31E−05)	1.533 (0.95, 2.51)	1.75 (8.02E−02)	0.950 (0.52, 1.69)	−0.17 (8.61E−01)

*The table shows the exact values for Figure 4. Due to the small sample size in this study, the covariates related to the disease status (e.g., hypertension, diabetes, and so on) were not included in the multiple logistic regression model. OR (95% CI), Odds ratios with 95% confident interval; Wald Z (*p*-value), *P*-value obtains from Wald test*.
